# Supplementary material for: Every animal matters! Evaluating the selectivity of a Mediterranean bottom trawl fishery from a species community perspective
Source: PLoS One. 2023 Mar 23;18(3):e0283362. doi: 10.1371/journal.pone.0283362 (PMC10035856; doi:10.1371/journal.pone.0283362)
Supplement: S1 Table — The green, yellow, orange and red areas represent the target species, the bycatch species of commercial value, the species of no commercial value and the protected species, respectively. (DOCX) [file pone.0283362.s001.docx]

**S1 Table. Dominance percentages of each species caught with the 50 mm diamond mesh codend (DM50) and the 40 mm square mesh codend (SM40), in both number of individuals and weight, for each fraction (total, discarded and landed).** The green, yellow, orange and red areas represent the target species, the bycatch species of commercial value, the species of no commercial value and the protected species, respectively.

| **Species**  **Rank** | **DM50** | | | | | | **SM40** | | | | | |
| --- | --- | --- | --- | --- | --- | --- | --- | --- | --- | --- | --- | --- |
|  | **Individuals** | | | **Weight** | | | **Individuals** | | | **Weight** | | |
|  | Total | Discarded | Landed | Total | Discarded | Landed | Total | Discarded | Landed | Total | Discarded | Landed |
| S1 | 2.6 (2.1 - 3.2) | 0.4 (0.2 - 0.6) | 23.1 (20.8 - 25.5) | 16.3 (14.0 - 18.9) | 1.0 (0.6 - 1.5) | 35.1 (30.7 - 39.5) | 3.6 (3.0 - 4.2) | 0.8 (0.6 - 1.0) | 19.7 (17.4 - 21.9) | 21.3 (17.5 - 24.8) | 2.0 (1.5 - 2.5) | 43.0 (36.7 - 48.4) |
| S2 | 0.7 (0.5- 0.9) | 0.0 (0.0 - 0.0) | 7.2 (5.6 - 9.0) | 1.4 (1.0 - 1.9) | 0.0 (0.0 - 0.0) | 3.1 (2.2 - 4.2) | 1.7 (1.3 - 2.1) | 0.0 (0.0 - 0.0) | 11.7 (9.5 - 14.0) | 2.9 (2.3 - 3.6) | 0.0 (0.0 - 0.0) | 6.2 (5.1 - 7.5) |
| S3 | 0.22 (0.1 - 0.3) | 0.0 (0.0 - 0.0) | 2.4 (1.5 - 3.3) | 7.5 (4.5 - 10.7) | 0.0 (0.0 - 0.0) | 16.6 (10.5 - 23.2) | 0.1 (0.0 - 0.2) | 0.0 (0.0 - 0.0) | 0.6 (0.3 - 1.1) | 2.6 (0.9 - 4.8) | 0.0 (0.0 - 0.0) | 5.6 (2.0 - 10.2) |
| S4 | 0.7 (0.5 - 0.9) | 0.0 (0.0 - 0.0) | 6.8 (5.0 - 8.6) | 3.9 (2.9 - 5.1) | 0.0 (0.0 - 0.0) | 8.6 (6.6 - 10.8) | 0.7 (0.6 - 0.9) | 0.0 (0.0 - 0.0) | 4.8 (4.0 - 5.7) | 4.2 (3.3 - 5.1) | 0.0 (0.0 - 0.0) | 8.9 (7.2 - 10.6) |
| S5 | 0.7 (0.5 - 1.0) | 0.0 (0.0 - 0.0) | 7.4 (5.8 - 9.3) | 1.1 (0.8 - 1.5) | 0.0 (0.0 - 0.0) | 2.6 (1.9 - 3.4) | 1.7 (1.4 - 2.2) | 0.0 (0.0 - 0.0) | 11.8 (10.1 - 13.9) | 2.6 (2.0 - 3.1) | 0.0 (0.0 - 0.0) | 5.4 (4.5 - 6.3) |
| S6 | 0.5 (0.2 - 0.8) | 0.0 (0.0 - 0.0) | 4.5 (2.3 - 7.1) | 2.2 (0.9 - 3.8) | 0.0 (0.0 - 0.1) | 4.9 (2.1 - 8.0) | 0.6 (0.3 - 1.1) | 0.0 (0.0 - 0.0) | 4.3 (1.8 - 7.7) | 2.9 (1.3 - 5.2) | 0.0 (0.0 - 0.0) | 6.1 (2.7 - 10.8) |
| S7 | 0.1 (0.0 - 0.1) | 0.1 (0.0 - 0.2) | 0.0 (0.0 - 0.0) | 0.1 (0.0 - 0.3) | 0.3 (0.0 - 0.6) | 0.0 (0.0 - 0.0) | 0.2 (0.1 - 0.3) | 0.2 (0.1 - 0.4) | 0.0 (0.0 - 0.0) | 0.2 (0.1 - 0.3) | 0.4 (0.2 - 0.7) | 0.0 (0.0 - 0.0) |
| S8 | 1.2 (0.7 - 1.9) | 0.0 (0.0 - 0.0) | 12.7 (9.2 - 17.3) | 0.6 (0.3 - 0.8) | 0.0 (0.0 - 0.0) | 1.3 (0.8 - 1.8) | 2.0 (1.3 - 2.9) | 0.0 (0.0 - 0.0) | 13.5 (9.7 - 18.8) | 0.9 (0.6 - 1.2) | 0.0 (0.0 - 0.0) | 1.8 (1.3 - 2.5) |
| S9 | 0.3 (0.1 - 0.4) | 0.0 (0.0 - 0.0) | 2.7 (1.5 - 4.1) | 1.5 (0.8 - 2.2) | 0.0 (0.0 - 0.0) | 3.2 (1.8 - 5.0) | 0.2 (0.1 - 0.4) | 0.0 (0.0 - 0.0) | 1.7 (0.9 - 2.6) | 1.3 (0.8 - 1.8) | 0.0 (0.0 - 0.0) | 2.7 (1.6 - 4.0) |
| S10 | 0.8 (0.5 - 1.2) | 0.0 (0.0 - 0.0) | 8.6 (4.7 - 13.9) | 3.7 (1.8 - 5.8) | 0.0 (0.0 - 0.0) | 8.1 (3.9 - 13.0) | 0.3 (0.2 - 0.4) | 0.0 (0.0 - 0.0) | 2.0 (1.1 - 3.0) | 1.6 (0.9 - 2.4) | 0.0 (0.0 - 0.0) | 3.4 (1.9 - 5.1) |
| S11 | 0.1 (0.0 - 0.1) | 0.0 (0.0 - 0.0) | 0.8 (0.3 - 1.5) | 1.8 (0.6 - 3.3) | 0.0 (0.0 - 0.0) | 3.9 (1.3 - 7.5) | 0.0 (0.0 - 0.0) | 0.0 (0.0 - 0.0) | 0.1 (0.0 - 0.3) | 0.3 (0.0 - 0.9) | 0.0 (0.0 - 0.0) | 0.7 (0.0 - 1.9) |
| S12 | 0.9 (0.6 - 1.4) | 0.0 (0.0 - 0.0) | 9.7 (7.2 - 12.2) | 1.1 (0.8 - 1.6) | 0.0 (0.0 - 0.0) | 2.5 (1.75 - 3.4) | 1.2 (0.9 - 1.6) | 0.0 (0.0 - 0.0) | 8.4 (6.7 - 10.3) | 1.4 (1.0 - 1.7) | 0.0 (0.0 - 0.0) | 2.9 (2.2 - 3.6) |
| S13 | 0.0 (0.0 - 0.0) | 0.0 (0.0 - 0.0) | 0.0 (0.0 - 0.0) | 0.0 (0.0 - 0.0) | 0.0 (0.0 - 0.0) | 0.0 (0.0 - 0.0) | 0.0 (0.0 - 0.0) | 0.0 (0.0 - 0.0) | 0.0 (0.0 - 0.2) | 0.1 (0.0 - 0.7) | 0.0 (0.0 - 0.0) | 0.3 (0.0 - 1.5) |
| S14 | 0.0 (0.0 - 0.0) | 0.0 (0.0 - 0.0) | 0.0 (0.0 - 0.0) | 0.0 (0.0 - 0.0) | 0.0 (0.0 - 0.0) | 0.0 (0.0 - 0.0) | 0.0 (0.0 - 0.1) | 0.0 (0.0 - 0.0) | 0.2 (0.0 - 0.6) | 0.1 (0.0 - 0.2) | 0.0 (0.0 - 0.0) | 0.1 (0.0 - 0.4) |
| S15 | 0.1 (0.0 - 0.2) | 0.0 (0.0 - 0.0) | 0.8 (0.3 - 1.5) | 2.2 (0.8 - 4.0) | 0.0 (0.0 - 0.0) | 4.9 (1.7 - 8.5) | 0.1 (0.1 - 0.2) | 0.0 (0.0 - 0.0) | 0.7 (0.3- 1.1) | 2.4 (1.2 - 3.9) | 0.0 (0.0 - 0.1) | 5.2 (2.5 - 8.2) |
| S16 | 0.0 (0.0 - 0.1) | 0.0 (0.0 - 0.1) | 0.0 (0.0 - 0.0) | 0.1 (0.0 - 0.3) | 0.2 (0.0 - 0.5) | 0.0 (0.0 - 0.0) | 0.0 (0.0 - 0.0) | 0.0 (0.0 - 0.0) | 0.0 (0.0 - 0.0) | 0.0 (0.0 - 0.0) | 0.0 (0.0 - 0.0) | 0.0 (0.0 - 0.0) |
| S17 | 0.0 (0.0 - 0.0) | 0.0 (0.0 - 0.0) | 0.0 (0.0 - 0.0) | 0.0 (0.0 - 0.1) | 0.0 (0.0 - 0.2) | 0.0 (0.0 - 0.0) | 0.0 (0.0 - 0.0) | 0.0 (0.0 - 0.0) | 0.0 (0.0 - 0.0) | 0.0 (0.0 - 0.0) | 0.0 (0.0 - 0.0) | 0.0 (0.0 - 0.0) |
| S18 | 0.6 (0.4 - 0.8) | 0.0 (0.0 - 0.0) | 6.3 (4.6 - 8.2) | 0.6 (0.5 - 0.8) | 0.0 (0.0 - 0.0) | 1.4 (1.0 - 1.8) | 2.4 (1.9 - 2.9) | 0.0 (0.0 - 0.0) | 16.1 (13.2 - 19.1) | 1.9 (1.5 - 2.2) | 0.0 (0.0 - 0.0) | 4.0 (3.2 - 4.8) |
| S19 | 1.8 (1.3 - 2.5) | 1.8 (1.3 - 2.6) | 1.7 (0.9 - 2.5) | 0.6 (0.4 - 0.8) | 0.8 (0.6 - 1.1) | 0.3 (0.2 - 0.5) | 5.6 (5.0 - 6.4) | 6.3 (5.6 - 7.3) | 1.3 (0.8 - 1.9) | 1.6 (1.4 - 1.9) | 2.8 (2.4 - 3.2) | 0.4 (0.2 - 0.5) |
| S20 | 0.0 (0.0 - 0.1) | 0.0 (0.0 - 0.0) | 0.2 (0.0 - 0.6) | 0.1 (0.0 - 0.3) | 0.0 (0.0 - 0.0) | 0.3 (0.0 - 0.7) | 0.0 (0.0 - 0.1) | 0.0 (0.0 - 0.0) | 0.3 (0.1 - 0.6) | 0.3 (0.0 - 0.6) | 0.0 (0.0 - 0.0) | 0.5 (0.1 - 1.2) |
| S21 | 0.0 (0.0 - 0.0) | 0.0 (0.0 - 0.0) | 0.0 (0.0 - 0.2) | 0.3 (0.0 - 1.3) | 0.0 (0.0 - 0.0) | 0.6 (0.1 - 2.7) | 0.0 (0.0 - 0.0) | 0.0 (0.0 - 0.0) | 0.1 (0.0 - 0.2) | 0.2 (0.0 - 0.6) | 0.0 (0.0 - 0.0) | 0.4 (0.0 - 1.2) |
| S22 | 0.0 (0.0 - 0.1) | 0.0 (0.0 - 0.0) | 0.1 (0.0 - 0.5) | 0.1 (0.0 - 0.2) | 0.0 (0.0 - 0.0) | 0.2 (0.0 - 0.5) | 0.0 (0.0- 0.1) | 0.0 (0.0 - 0.0) | 0.1 (0.0 - 0.4) | 0.2 (0.0 - 0.6) | 0.0 (0.0 - 0.0) | 0.4 (0.0 - 1.2) |
| S23 | 0.1 (0.0 - 0.2) | 0.0 (0.0 - 0.0) | 0.7 (0.1 - 1.6) | 0.1 (0.0 - 0.2) | 0.0 (0.0 - 0.0) | 0.3 (0.1 - 0.5) | 0.1 (0.0- 0.1) | 0.0 (0.0 - 0.0) | 0.4 (0.1 - 0.7) | 0.1 (0.0 - 0.2) | 0.0 (0.0 - 0.0) | 0.2 (0.0 - 0.4) |
| S24 | 0.4 (0.2 - 0.6) | 0.0 (0.0 - 0.1) | 3.7 (1.8 - 6.1) | 0.7 (0.4 - 1.1) | 0.0 (0.0 - 0.1) | 1.5 (0.8 - 2.4) | 0.2 (0.1 - 0.3) | 0.0 (0.0 - 0.0) | 1.3 (0.7 - 2.3) | 0.4 (0.2 - 0.6) | 0.0 (0.0 - 0.0) | 0.8 (0.4 - 1.4) |
| S25 | 0.1 (0.0 - 0.2) | 0.1 (0.0 - 0.3) | 0.0 (0.0 - 0.0) | 0.0 (0.0 - 0.1) | 0.1 (0.0 - 0.2) | 0.0 (0.0 - 0.0) | 0.3 (0.1 - 0.4) | 0.3 (0.1 - 0.5) | 0.0 (0.0 - 0.0) | 0.1 (0.0 - 0.1) | 0.1 (0.1 - 0.2) | 0.0 (0.0 - 0.0) |
| S26 | 0.0 (0.0 - 0.0) | 0.0 (0.0 - 0.0) | 0.0 (0.0 - 0.0) | 0.0 (0.0 - 0.1) | 0.0 (0.0 - 0.1) | 0.0 (0.0 - 0.0) | 0.0 (0.0 - 0.0) | 0.0 (0.0 - 0.0) | 0.0 (0.0 - 0.0) | 0.0 (0.0 - 0.0) | 0.0 (0.0 - 0.0) | 0.0 (0.0 - 0.0) |
| S27 | 0.0 (0.0 - 0.0) | 0.0 (0.0 - 0.0) | 0.1 (0.0 - 0.3) | 0.1 (0.0 - 0.3) | 0.0 (0.0 - 0.0) | 0.2 (0.0 - 0.7) | 0.0 (0.0 - 0.1) | 0.0 (0.0 - 0.0) | 0.2 (0.0 - 0.4) | 0.2 (0.0 - 0.4) | 0.0 (0.0 - 0.1) | 0.3 (0.0 - 0.9) |
| S28 | 0.0 (0.0 - 0.1) | 0.0 (0.0 - 0.0) | 0.2 (0.0 - 0.5) | 0.1 (0.0 - 0.3) | 0.0 (0.0 - 0.0) | 0.2 (0.0 - 0.6) | 0.1 (0.0 - 0.1) | 0.0 (0.0 - 0.0) | 0.4 (0.1 - 0.8) | 0.2 (0.1 - 0.5) | 0.0 (0.0 - 0.1) | 0.5 (0.1 - 1.0) |
| S29 | 0.0 (0.0 - 0.0) | 0.0 (0.0 - 0.0) | 0.0 (0.0 - 0.2) | 0.0 (0.0 - 0.1) | 0.0 (0.0 - 0.1) | 0.1 (0.0 - 0.3) | 0.0 (0.0 - 0.1) | 0.0 (0.0 - 0.0) | 0.2 (0.0 - 0.4) | 0.1 (0.0 - 0.2) | 0.0 (0.0 - 0.0) | 0.2 (0.0 - 0.5) |
| S30 | 0.0 (0.0 - 0.0) | 0.0 (0.0 - 0.0) | 0.0 (0.0 - 0.2) | 0.0 (0.0 - 0.2) | 0.0 (0.0 - 0.0) | 0.1 (0.0 - 0.5) | 0.0 (0.0 - 0.0) | 0.0 (0.0 - 0.0) | 0.0 (0.0 - 0.0) | 0.0 (0.0 - 0.0) | 0.0 (0.0 - 0.0) | 0.0 (0.0 - 0.0) |
| S31 | 0.0 (0.0 - 0.0) | 0.0 (0.0 - 0.0) | 0.0 (0.0 - 0.0) | 0.0 (0.0 - 0.0) | 0.0 (0.0 - 0.0) | 0.0 (0.0 - 0.0) | 0.0 (0.0 - 0.0) | 0.0 (0.0 - 0.0) | 0.0 (0.0 - 0.2) | 0.0 (0.0 - 0.2) | 0.0 (0.0 - 0.0) | 0.1 (0.0 - 0.4) |
| S32 | 80.1 (75.7 - 83.5) | 88.7 (85.1 - 91.4) | 0.0 (0.0 - 0.0) | 42.7 (38.3 - 47.0) | 77.7 (73.7 - 81.9) | 0.0 (0.0 - 0.0) | 73.8 (70.9 - 76.5) | 86.5 (84.1 - 88.6) | 0.0 (0.0 - 0.0) | 42.9 (38.1 - 47.6) | 81.2 (74.9 - 85.8) | 0.0 (0.0 - 0.0) |
| S33 | 0.5 (0.3 - 0.7) | 0.5 (0.3 - 0.7) | 0.0 (0.0 - 0.0) | 0.4 (0.3 - 0.6) | 0.8 (0.5 - 1.2) | 0.0 (0.0 - 0.0) | 0.3 (0.2 - 0.5) | 0.3 (0.2 - 0.5) | 0.0 (0.0 - 0.0) | 0.2 (0.2 - 0.4) | 0.5 (0.3 - 0.7) | 0.0 (0.0 - 0.0) |
| S34 | 0.1 (0.0 - 0.2) | 0.1 (0.0 - 0.2) | 0.0 (0.0 - 0.0) | 0.0 (0.0 - 0.1) | 0.0 (0.0 - 0.1) | 0.0 (0.0 - 0.0) | 0.1 (0.0 - 0.2) | 0.1 (0.0 - 0.2) | 0.0 (0.0 - 0.0) | 0.0 (0.0 - 0.1) | 0.1 (0.0 - 0.2) | 0.0 (0.0 - 0.0) |
| S35 | 0.0 (0.0 - 0.0) | 0.0 (0.0 - 0.0) | 0.0 (0.0 - 0.0) | 0.0 (0.0 - 0.0) | 0.0 (0.0 - 0.0) | 0.0 (0.0 - 0.0) | 0.0 (0.0 - 0.0) | 0.0 (0.0 - 0.0) | 0.0 (0.0 - 0.0) | 0.0 (0.0 - 0.1) | 0.0 (0.0 - 0.1) | 0.0 (0.0 - 0.0) |
| S36 | 0.0 (0.0 - 0.0) | 0.0 (0.0 - 0.0) | 0.0 (0.0 - 0.0) | 0.1 (0.0 - 0.4) | 0.2 (0.0 - 0.7) | 0.0 (0.0 - 0.0) | 0.0 (0.0 - 0.0) | 0.0 (0.0 - 0.0) | 0.0 (0.0 - 0.0) | 0.0 (0.0 - 0.0) | 0.0 (0.0 - 0.0) | 0.0 (0.0 - 0.0) |
| S37 | 0.0 (0.0 - 0.0) | 0.0 (0.0 - 0.0) | 0.0 (0.0 - 0.0) | 0.0 (0.0 - 0.0) | 0.0 (0.0 - 0.0) | 0.0 (0.0 - 0.0) | 0.0 (0.0 - 0.0) | 0.0 (0.0 - 0.0) | 0.0 (0.0 - 0.0) | 0.0 (0.0 - 0.0) | 0.0 (0.0 - 0.0) | 0.0 (0.0 - 0.0) |
| S38 | 2.7 (1.7 - 3.7) | 3.0 (1.9 - 4.1) | 0.0 (0.0 - 0.0) | 1.8 (1.1- 2.4) | 3.3 (2.1 - 4.4) | 0.0 (0.0 - 0.0) | 1.3 (0.6 - 2.1) | 1.5 (0.7 - 2.5) | 0.0 (0.0 - 0.0) | 0.9 (0.4 - 1.5) | 1.7 (0.8 - 3.0) | 0.0 (0.0 - 0.0) |
| S39 | 1.3 (0.1 - 3.0) | 1.5 (0.1 - 3.4) | 0.0 (0.0 - 0.0) | 0.5 (0.1 - 1.0) | 0.9 (0.1 - 1.9) | 0.0 (0.0 - 0.0) | 0.8 (0.3- 1.6) | 1.0 (0.3 - 1.9) | 0.0 (0.0 - 0.0) | 0.3 (0.1 - 0.6) | 0.6 (0.1 - 1.1) | 0.0 (0.0 - 0.0) |
| S40 | 0.0 (0.0 - 0.1) | 0.0 (0.0 - 0.1) | 0.0 (0.0 - 0.0) | 0.0 (0.0 - 0.1) | 0.1 (0.0- 0.2) | 0.0 (0.0 - 0.0) | 0.0 (0.0 - 0.0) | 0.0 (0.0 - 0.0) | 0.0 (0.0 - 0.0) | 0.0 (0.0 - 0.1) | 0.0 (0.0 - 0.1) | 0.0 (0.0 - 0.0) |
| S41 | 0.0 (0.0 - 0.0) | 0.0 (0.0 - 0.1) | 0.0 (0.0 - 0.0) | 0.0 (0.0 - 0.0) | 0.0 (0.0 - 0.1) | 0.0 (0.0 - 0.0) | 0.0 (0.0 - 0.1) | 0.0 (0.0 - 0.1) | 0.0 (0.0 - 0.0) | 0.0 (0.0 - 0.1) | 0.1 (0.0 - 0.1) | 0.0 (0.0 - 0.0) |
| S42 | 0.0 (0.0 - 0.0) | 0.0 (0.0 - 0.1) | 0.0 (0.0 - 0.0) | 0.0 (0.0 - 0.1) | 0.0 (0.0 - 0.1) | 0.0 (0.0 - 0.0) | 0.0 (0.0 - 0.1) | 0.0 (0.0 - 0.1) | 0.0 (0.0 - 0.0) | 0.1 (0.0- 0.2) | 0.1 (0.0 - 0.3) | 0.0 (0.0 - 0.0) |
| S43 | 0.1 (0.0 - 0.2) | 0.1 (0.0 - 0.2) | 0.0 (0.0 - 0.0) | 0.2 (0.1 - 0.4) | 0.4 (0.1 - 0.7) | 0.0 (0.0 - 0.0) | 0.2 (0.1 - 0.3) | 0.2 (0.1 - 0.3) | 0.0 (0.0 - 0.0) | 0.3 (0.1 - 0.5) | 0.6 (0.3 - 0.9) | 0.0 (0.0 - 0.0) |
| S44 | 0.0 (0.0 - 0.0) | 0.0 (0.0 - 0.0) | 0.0 (0.0 - 0.0) | 0.1 (0.0 - 0.2) | 0.1 (0.0 - 0.4) | 0.0 (0.0 - 0.0) | 0.0 (0.0 - 0.0) | 0.0 (0.0 - 0.0) | 0.0 (0.0 - 0.0) | 0.7 (0.0- 3.5) | 1.4 (0.0 - 6.5) | 0.0 (0.0 - 0.0) |
| S45 | 0.46 (0.3 - 0.7) | 0.5 (0.3 - 0.8) | 0.0 (0.0 - 0.0) | 0.6 (0.3 - 0.8) | 1.0 (0.6 - 1.5) | 0.0 (0.0 - 0.0) | 0.3 (0.2 - 0.4) | 0.3 (0.2 - 0.5) | 0.0 (0.0 - 0.0) | 0.4 (0.2 - 0.5) | 0.7 (0.4 - 0.9) | 0.0 (0.0 - 0.0) |
| S46 | 0.0 (0.0 - 0.1) | 0.0 (0.0 - 0.1) | 0.0 (0.0 - 0.0) | 0.0 (0.0 - 0.1) | 0.1 (0.0 - 0.2) | 0.0 (0.0 - 0.0) | 0.1 (0.0 - 0.2) | 0.1 (0.1 - 0.2) | 0.0 (0.0 - 0.0) | 0.1 (0.1 - 0.2) | 0.2 (0.1 - 0.4) | 0.0 (0.0 - 0.0) |
| S47 | 0.0 (0.0 - 0.0) | 0.0 (0.0 - 0.0) | 0.0 (0.0 - 0.0) | 0.0 (0.0 - 0.0) | 0.0 (0.0 - 0.0) | 0.0 (0.0 - 0.0) | 0.0 (0.0 - 0.0) | 0.0 (0.0 - 0.0) | 0.0 (0.0 - 0.0) | 0.0 (0.0 - 0.0) | 0.0 (0.0 - 0.0) | 0.0 (0.0 - 0.0) |
| S48 | 0.0 (0.0 - 0.1) | 0.0 (0.0 - 0.1) | 0.0 (0.0 - 0.0) | 0.0 (0.0 - 0.0) | 0.0 (0.0 - 0.0) | 0.0 (0.0 - 0.0) | 0.0 (0.0 - 0.1) | 0.0 (0.0 - 0.1) | 0.0 (0.0 - 0.0) | 0.0 (0.0 - 0.0) | 0.0 (0.0 - 0.0) | 0.0 (0.0 - 0.0) |
| S49 | 0.0 (0.0 - 0.0) | 0.0 (0.0 - 0.0) | 0.0 (0.0 - 0.0) | 0.0 (0.0 - 0.0) | 0.0 (0.0 - 0.0) | 0.0 (0.0 - 0.0) | 0.0 (0.0 - 0.1) | 0.0 (0.0 - 0.1) | 0.0 (0.0 - 0.0) | 0.0 (0.0 - 0.0) | 0.0 (0.0 - 0.1) | 0.0 (0.0 - 0.0) |
| S50 | 0.0 (0.0 - 0.1) | 0.1 (0.0 - 0.2) | 0.0 (0.0 - 0.0) | 0.1 (0.0 - 0.2) | 0.1 (0.0 - 0.5) | 0.0 (0.0 - 0.0) | 0.0 (0.0 - 0.0) | 0.0 (0.0 - 0.0) | 0.0 (0.0 - 0.0) | 0.0 (0.0 - 0.0) | 0.0 (0.0 - 0.0) | 0.0 (0.0 - 0.0) |
| S51 | 0.0 (0.0 - 0.1) | 0.0 (0.0 - 0.1) | 0.0 (0.0 - 0.0) | 0.0 (0.0 - 0.2) | 0.1 (0.0 - 0.3) | 0.0 (0.0 - 0.0) | 0.0 (0.0 - 0.0) | 0.0 (0.0 - 0.0) | 0.0 (0.0 - 0.0) | 0.0 (0.0 - 0.0) | 0.0 (0.0 - 0.0) | 0.0 (0.0 - 0.0) |
| S52 | 0.1 (0.0 - 0.3) | 0.1 (0.0 - 0.4) | 0.0 (0.0 - 0.0) | 0.2 (0.0 - 0.7) | 0.4 (0.0 - 1.4) | 0.0 (0.0 - 0.0) | 0.0 (0.0- 0.1) | 0.1 (0.0 - 0.1) | 0.0 (0.0 - 0.0) | 0.0 (0.0 - 0.1) | 0.1 (0.0 - 0.2) | 0.0 (0.0 - 0.0) |
| S53 | 0.0 (0.0 - 0.0) | 0.0 (0.0 - 0.0) | 0.0 (0.0 - 0.0) | 0.0 (0.0 - 0.1) | 0.0 (0.0 - 0.1) | 0.0 (0.0 - 0.0) | 0.0 (0.0 - 0.0) | 0.0 (0.0 - 0.0) | 0.0 (0.0 - 0.0) | 0.0 (0.0 - 0.0) | 0.0 (0.0 - 0.0) | 0.0 (0.0 - 0.0) |
| S54 | 0.0 (0.0 - 0.0) | 0.0 (0.0 - 0.0) | 0.0 (0.0 - 0.0) | 0.0 (0.0 - 0.0) | 0.0 (0.0 - 0.0) | 0.0 (0.0 - 0.0) | 0.0 (0.0 - 0.0) | 0.0 (0.0 - 0.0) | 0.0 (0.0 - 0.0) | 0.0 (0.0 - 0.0) | 0.0 (0.0 - 0.0) | 0.0 (0.0 - 0.0) |
| S55 | 0.1 (0.1 - 0.3) | 0.1 (0.1 - 0.3) | 0.0 (0.0 - 0.0) | 0.1 (0.0 - 0.2) | 0.2 (0.1 - 0.3) | 0.0 (0.0 - 0.0) | 0.0 (0.0 - 0.1) | 0.1 (0.0 - 0.2) | 0.0 (0.0 - 0.0) | 0.1 (0.0 - 0.1) | 0.1 (0.0 - 0.2) | 0.0 (0.0 - 0.0) |
| S56 | 0.0 (0.0 - 0.1) | 0.0 (0.0 - 0.1) | 0.0 (0.0 - 0.0) | 0.1 (0.0 - 0.1) | 0.1 (0.0 - 0.3) | 0.0 (0.0 - 0.0) | 0.3 (0.0 - 0.6) | 0.3 (0.0 - 0.7) | 0.0 (0.0 - 0.0) | 0.4 (0.0- 0.9) | 0.8 (0.1 - 1.9) | 0.0 (0.0 - 0.0) |
| S57 | 0.1 (0.1 - 0.3) | 0.2 (0.1 - 0.3) | 0.0 (0.0 - 0.0) | 0.1 (0.1 - 0.2) | 0.2 (0.1 - 0.3) | 0.0 (0.0 - 0.0) | 0.5 (0.3 - 0.7) | 0.6 (0.4 - 0.8) | 0.0 (0.0 - 0.0) | 0.4 (0.2 - 0.5) | 0.7 (0.4 - 1.1) | 0.0 (0.0 - 0.0) |
| S58 | 0.0 (0.0 - 0.0) | 0.0 (0.0 - 0.0) | 0.0 (0.0 - 0.0) | 0.0 (0.0 - 0.1) | 0.0 (0.0 - 0.2) | 0.0 (0.0 - 0.0) | 0.0 (0.0 - 0.0) | 0.0 (0.0 - 0.0) | 0.0 (0.0 - 0.0) | 0.0 (0.0 - 0.1) | 0.0 (0.0 - 0.2) | 0.0 (0.0 - 0.0) |
| S59 | 0.4 (0.2 - 0.6) | 0.4 (0.2 - 0.7) | 0.0 (0.0 - 0.0) | 1.5 (0.7 - 2.3) | 2.7 (1.3 - 4.1) | 0.0 (0.0 - 0.0) | 0.1 (0.1 - 0.2) | 0.1 (0.1 - 0.2) | 0.0 (0.0 - 0.0) | 0.4 (0.2 - 0.6) | 0.7 (0.3 - 1.2) | 0.0 (0.0 - 0.0) |
| S60 | 1.2 (0.7 - 1.8) | 1.3 (0.7 - 1.9) | 0.0 (0.0 - 0.0) | 1.7 (0.9 - 2.5) | 3.0 (1.6 - 4.5) | 0.0 (0.0 - 0.0) | 0.2 (0.1 - 0.4) | 0.3 (0.1 - 0.4) | 0.0 (0.0 - 0.0) | 0.3 (0.1 - 0.5) | 0.5 (0.2 - 1.0) | 0.0 (0.0 - 0.0) |
| S61 | 0.3 (0.2 - 0.4) | 0.3 (0.2 - 0.4) | 0.0 (0.0 - 0.0) | 2.4 (1.3 - 3.4) | 4.3 (2.5 - 6.1) | 0.0 (0.0 - 0.0) | 0.1 (0.1 - 0.2) | 0.1 (0.1 - 0.2) | 0.0 (0.0 - 0.0) | 1.1 (0.5 - 1.8) | 2.1 (1.0 - 3.4) | 0.0 (0.0 - 0.0) |
| S62 | 0.0 (0.0 - 0.0) | 0.0 (0.0 - 0.0) | 0.0 (0.0 - 0.0) | 0.1 (0.0 - 0.3) | 0.1 (0.0 - 0.6) | 0.0 (0.0 - 0.0) | 0.0 (0.0 - 0.0) | 0.0 (0.0 - 0.0) | 0.0 (0.0 - 0.0) | 0.0 (0.0 - 0.0) | 0.0 (0.0 - 0.0) | 0.0 (0.0 - 0.0) |
| S63 | 0.2 (0.1 - 0.3) | 0.2 (0.1 - 0.3) | 0.0 (0.0 - 0.0) | 0.4 (0.2 - 0.8) | 0.8 (0.3 - 1.4) | 0.0 (0.0 - 0.0) | 0.1 (0.0 - 0.1) | 0.1 (0.0 - 0.2) | 0.0 (0.0 - 0.0) | 0.2 (0.1 - 0.4) | 0.4 (0.1 - 0.8) | 0.0 (0.0 - 0.0) |
| S64 | 0.0 (0.0 - 0.0) | 0.0 (0.0 - 0.0) | 0.0 (0.0 - 0.0) | 0.0 (0.0 - 0.0) | 0.0 (0.0 - 0.0) | 0.0 (0.0 - 0.0) | 0.0 (0.0 - 0.0) | 0.0 (0.0 - 0.0) | 0.0 (0.0 - 0.0) | 0.0 (0.0 - 0.0) | 0.0 (0.0 - 0.0) | 0.0 (0.0 - 0.0) |
| S65 | 0.0 (0.0 - 0.0) | 0.0 (0.0 - 0.0) | 0.0 (0.0 - 0.0) | 0.0 (0.0 - 0.0) | 0.0 (0.0 - 0.0) | 0.0 (0.0 - 0.0) | 0.0 (-0.0 - 0.1) | 0.0 (0.0 - 0.1) | 0.0 (0.0 - 0.0) | 0.2 (0.0 - 0.6) | 0.4 (0.0 - 1.2) | 0.0 (0.0 - 0.0) |
| S66 | 0.0 (0.0 - 0.0) | 0.0 (0.0 - 0.0) | 0.0 (0.0 - 0.0) | 0.0 (0.0 - 0.0) | 0.0 (0.0 - 0.0) | 0.0 (0.0 - 0.0) | 0.3 (0.0 - 0.9) | 0.3 (0.0 - 1.1) | 0.0 (0.0 - 0.0) | 0.5 (0.0 - 1.7) | 1.0 (0.0 - 3.2) | 0.0 (0.0 - 0.0) |
| S67 | 0.0 (0.0 - 0.1) | 0.0 (0.0 - 0.1) | 0.0 (0.0 - 0.0) | 0.5 (0.1 - 1.0) | 0.9 (0.1 - 1.8) | 0.0 (0.0 - 0.0) | 0.0 (0.0 - 0.0) | 0.0 (0.0 - 0.1) | 0.0 (0.0 - 0.0) | 0.2 (0.0 - 0.5) | 0.3 (0.0 - 1.0) | 0.0 (0.0 - 0.0) |
| S68 | 0.0 (0.0 - 0.0) | 0.0 (0.0 - 0.0) | 0.0 (0.0 - 0.0) | 0.0 (0.0 - 0.1) | 0.0 (0.0 - 0.2) | 0.0 (0.0 - 0.0) | 0.0 (0.0 - 0.1) | 0.0 (0.0 - 0.1) | 0.0 (0.0 - 0.0) | 0.2 (0.0 - 0.6) | 0.4 (0.0 - 1.2) | 0.0 (0.0 - 0.0) |
